# Cancer Incidence in Egypt: Results of the National Population-Based Cancer Registry Program

**DOI:** 10.1155/2014/437971

**Published:** 2014-09-21

**Authors:** Amal S. Ibrahim, Hussein M. Khaled, Nabiel NH Mikhail, Hoda Baraka, Hossam Kamel

**Affiliations:** ^1^Department of Biostatistics and Cancer Epidemiology, National Cancer Institute, Cairo University, Cairo, Egypt; ^2^Department of Medical Oncology, National Cancer Institute, Cairo University, Cairo, Egypt; ^3^Department of Biostatistics and Cancer Epidemiology, South Egypt Cancer Institute, Assiut University, Assiut, Egypt; ^4^Department of Computer Engineering, Faculty of Engineering, Cairo University, Giza, Egypt

## Abstract

*Background*. This paper aims to present cancer incidence rates at national and regional level of Egypt, based upon results of National Cancer Registry Program (NCRP). *Methods*. NCRP stratified Egypt into 3 geographical strata: lower, middle, and upper. One governorate represented each region. Abstractors collected data from medical records of cancer centers, national tertiary care institutions, Health Insurance Organization, Government-Subsidized Treatment Program, and death records. Data entry was online. Incidence rates were calculated at a regional and a national level. Future projection up to 2050 was also calculated. *Results*. Age-standardized incidence rates per 100,000 were 166.6 (both sexes), 175.9 (males), and 157.0 (females). Commonest sites were liver (23.8%), breast (15.4%), and bladder (6.9%) (both sexes): liver (33.6%) and bladder (10.7%) among men, and breast (32.0%) and liver (13.5%) among women. By 2050, a 3-fold increase in incident cancer relative to 2013 was estimated. *Conclusion*. These data are the only available cancer rates at national and regional levels of Egypt. The pattern of cancer indicated the increased burden of liver cancer. Breast cancer occupied the second rank. Study of rates of individual sites of cancer might help in giving clues for preventive programs.

## 1. Introduction

Egypt was completely lacking incidence rates at national level until the results given in the current report were obtained. Available statistics were proportions derived from single or multicenter hospital registries that could not be used for calculation of incidence rates [1–7]. The only published incidence rates are those from a cancer registry in one district in Nile delta (Gharbiah governorate). The last internal reports of this registry are for 2002 [8, 9]. Incidence rates up to 2007 were published in Volumes IX and X of Cancer Incidence in Five Continents [10, 11], date of end of registry activities due to failure of sustainability. The published crude and age-standardized incidence rates from that registry are 96.5 and 132.6/100,000 males and 97.3 and 122.1/100,000 females. The commonest sites of cancer in males are liver (18.7%), bladder (12.7%), non-Hodgkin's lymphoma (11.0%) and trachea, bronchus, and lung (8.2%). The 4 sites represent 50.6% of all cancer in males. The commonest sites in females are breast (38.8%), non-Hodgkin's lymphoma (8.5%), liver (4.6%), and ovary (4.5%); all together represent 56.4% of cancer in females. There is no mention of rates of both sexes together. Studies are published using these Gharbiah data up to 2007 and are limited to specific sites of cancer mainly breast [12–15], gastrointestinal [16–18], hematopoietic [19], bladder [20], and gynecological cancers [21]. None of these geographically-limited studies and published rates could be considered as representative of Egypt, being based on results of one registry in a single delta governorate and do not have an impact on understanding the current situation of cancer at the national level.

The National Cancer Registry Program (NCRP) was established in 2008 and became the only source for cancer incidence in the country [22]. The main objective of the current publication is to present the incidence rates of cancer in Egypt in 2008–2011 based upon data of the National Cancer Registry Program of Egypt with estimated incidence of the disease up to 2050.

## 2. Materials and Methods

**Figure 1:**
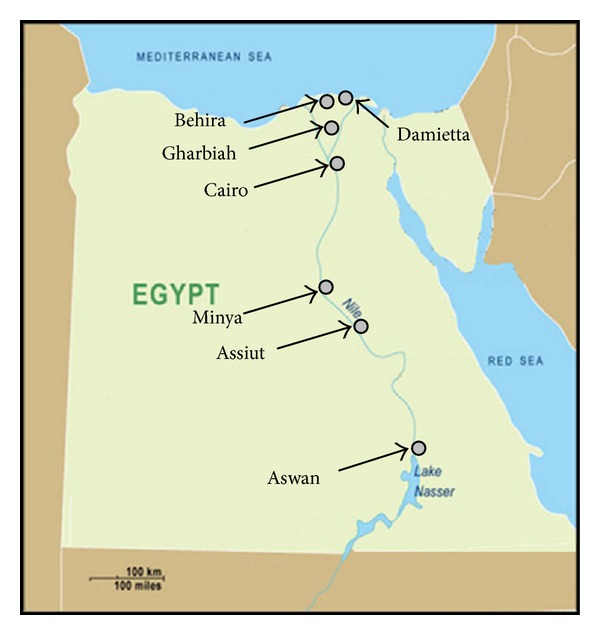
Map of Egypt showing the location of the 5 peripheral registries of the national network of population-based cancer registries National Cancer Institute, Cairo, and South Egypt Cancer Institute, Assiut.

For registration purposes, Egypt was stratified into 3 geographical strata (regions), namely, Lower Egypt (north of Cairo), Middle Egypt (south of Cairo), and Upper Egypt (further south, reaching the southern frontier of the country). The current report covered three districts (governorate), each representing one of the 3 regions, namely, Damietta (Nile delta), Minya (Middle Egypt), and Aswan (Upper Egypt) (Figure 1). A population-based registry was established in each of the 3 governorates, located in the Ministry of Health Cancer Center.

Trained medical doctors in the 3 registries abstracted records from their cancer centers and regularly visited establishments that dealt with cancer within the governorate for active data collection from medical records. Other sources of data were major tertiary centers on the national level as the National Cancer Institute of Cairo University, Pediatric Oncology Hospital in Cairo, and South Egypt Cancer Institute of Assiut University. Data managers in these institutions reported incident cancer cases among residents of the 3 governorates that were diagnosed/treated in these institutions. Health Insurance Organization and the Government-Subsidized Treatment Program periodically supplied their data. Death registers in local health directorates were regularly checked for cancer deaths from the 3 governorates.

**Figure 2:**
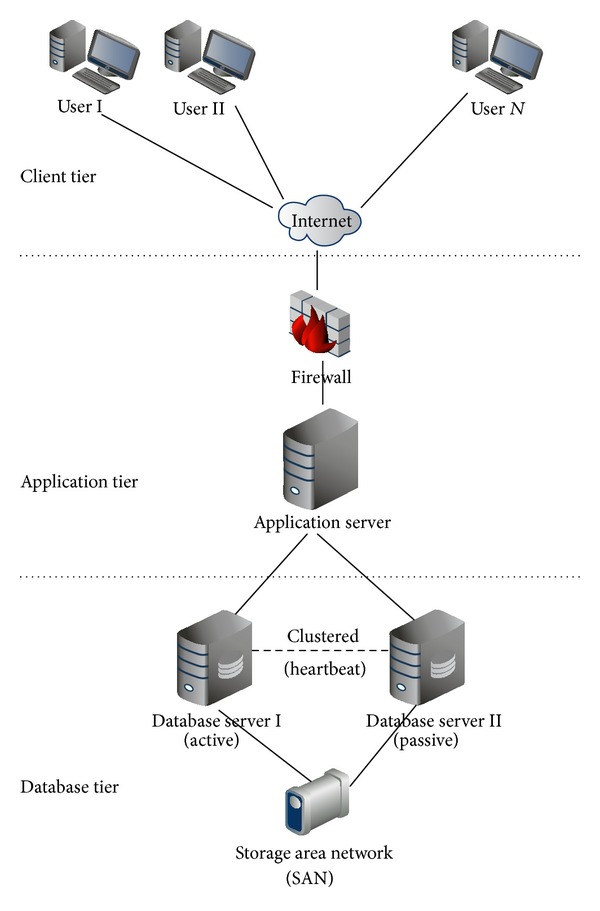
The NCRP Web Based System (3-Tier Architecture).

A web-based software was developed for online data entry, validity checks, and data analysis. The database was centralized in the Ministry of Communication and Information Technology server (Figure 2) with backups in the Ministry of Health and the National Cancer Institute. Computer checks were achieved using DEPedits Conversion and Check Programs for Cancer Registries software [23]. Duplicates were eliminated using the National Identification Number and a clean database was achieved ready for analysis. Registration covered all invasive cancers (behavior code/3), in situ breast cancer (topography code C50._ and behavior code/2), in situ urinary bladder cancer (topography code C67._ and behavior code/2), and borderline tumors of the brain (topography code C71._ and behavior code/1) [24].

For the current report, we used data from Aswan (2008), Minya (2009), and Damietta (2009–2011) to represent the 3 geographical strata of Egypt (Table 1). Crude, age-specific, and world population age-standardized incidence rates were calculated and expressed/100,000 population. Statistics were published at the level of the 3-character ICD-10 codes [10]. The format of IARC publication: “Cancer Incidence in Five Continents, Vol. X” [11], was used for grouping of ICD codes. Statistics for all sites of cancer were expressed twice, with and without nonmelanoma skin cancer (C44). Confidence intervals were calculated following SEER methodology [25].

We developed a model to use incidence data of the population-based registries of the 3 regions (Lower, Middle, and Upper Egypt) to get incidence rates for the entire country. The first step was to apply the age-specific incidence rates of each registry to the population of the stratum it represented to get the number per gender of incident cases in different age groups and for all ages together in this region using data of Egypt last census [26]. Then, we used these statistics to estimate the crude and age specific incidence rates (ASIR) of cancer in the 3 regions together, considered to be representative of the entire country. These rates were applied to the total population of Egypt to get number of incident cancers and crude rate and ASR (world) at national. This procedure was applied for each cancer site and for all sites together per gender. For projections up to 2050, the ASIRs were applied to the projected population of the corresponding year to get the number of patients/age group. Crude and ASR (world) could then be obtained. Steps of calculations are detailed in the Appendix.

## 3. Results

### 3.1. National Incidence Rates and Proportions

**Figure 3:**
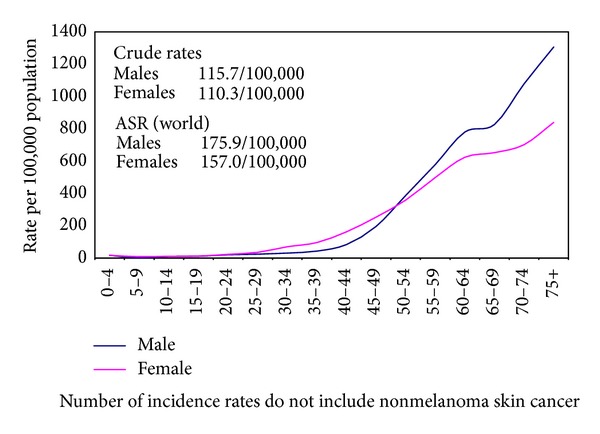
Calculated age specific incidence rates for Egypt 2008–2011.

**Figure 4:**
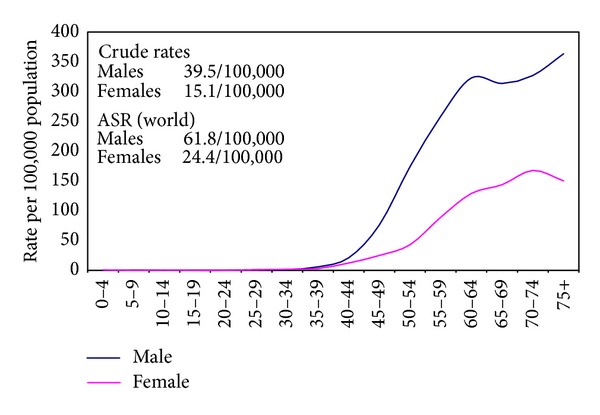
Calculated age specific incidence rates for liver cancer in Egypt 2008–2011.

**Figure 5:**
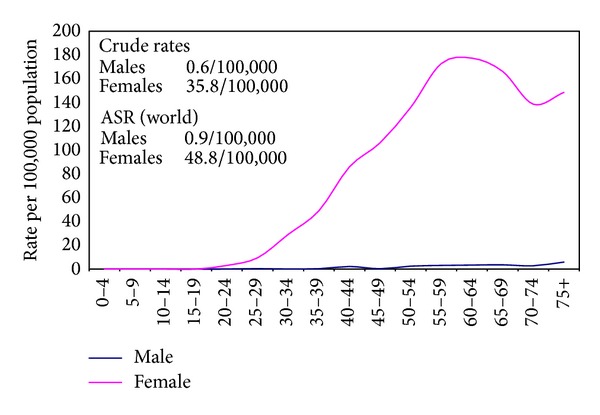
Calculated age specific incidence rates for breast cancer in Egypt 2008–2011.

**Figure 6:**
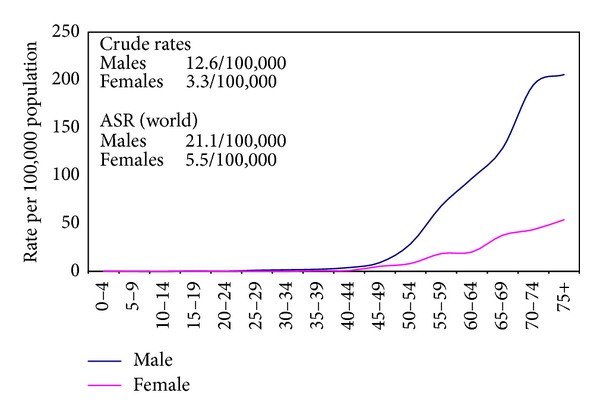
Calculated age specific incidence rates for urinary bladder cancer in Egypt 2008–2011.

The crude incidence rates on the national level for all sites excluding nonmelanoma skin cancer (C44) were 113.1/100,000 (both sexes), 115.7/100,000 (males), and 110.3/100,000 (females). The age-standardized rates (world) were 166.6/100,000 (both sexes), 175.9/100,000 (males), and 157.0/100,000 (females) as shown in Table 2. Proportions, crude, age standardized incidence rates and detailed age-specific rates of cancer sites according to ICD-10 format are shown in Tables 3 and 4 for individual sites of cancer and for all sites together by gender. The age-specific rates are represented graphically in Figure 3 for all cancers and Figures 4, 5, and 6 for breast, liver, and bladder cancer as examples of some of the more frequent cancer sites.

Table 5 depicts the proportions and rates of the most frequent cancer sites by gender. There was predominance of liver, breast, and bladder cancer that represented approximately 46% of all cancers. Liver and bladder cancers represented approximately 44% of cancer in males. In females, breast and liver cancer occupied the top ranks accounting for around 45% of all cancers.

### 3.2. Frequencies and Incidence Rates/Geographical Strata

The frequencies of individual sites of cancer and their incidence rates by geographical stratum and sex are detailed in Tables 6 and 7.  Table 8 depicts the most common sites of cancer that accounted for approximately 3/4 of cases. For the 2 sexes together, the top 2 ranks in the 3 regions were liver and breast cancer. The proportions and ASR of liver cancer were highest in Lower Egypt (29.6% and 56.8/100,000), less in Middle, and least in Upper Egypt (8.2% and 13.1/100,000).

Among males in the 3 regions, liver and bladder cancer occupied the top 2 ranks. The proportion and ASR of liver cancer were highest in Lower Egypt (41.7% and 81.0/100,000) and lowest in Upper Egypt (11.8% and 17.5/100,000). Cancer of the lung occupied the third or fourth ranks representing 5–7% of cancers and Non-Hodgkin lymphoma was among the 5 most common cancers in Lower Egypt only having a proportion of 6.0% and ASR 10.3/100,000.

Among females, the pattern in the 3 regions was dominated by the high frequency of breast cancer and liver cancer. Proportion of liver cancer was highest in Lower Egypt (16.4%), less in Middle Egypt, and lowest in Upper Egypt (8.9% and 5.1% resp.).

### 3.3. Estimated Number of Incident Cancer Cases 2013–2050

**Figure 7:**
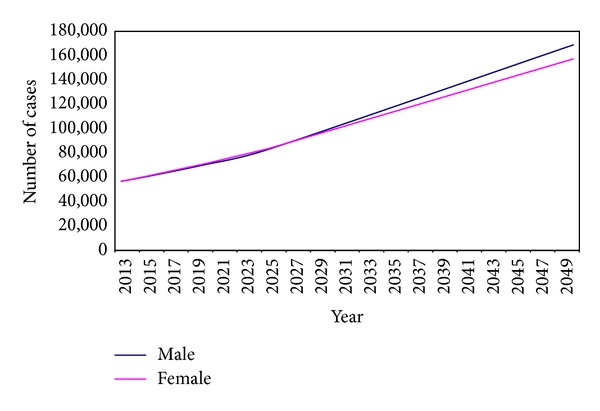
Estimated number of cases in Egypt (2013–2050).

**Figure 8:**
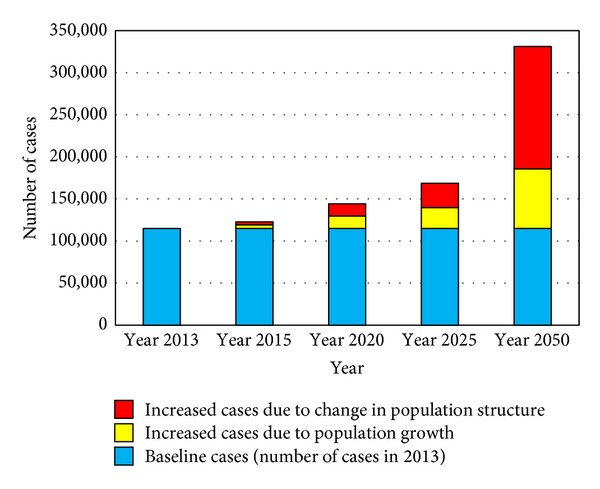
Estimated number of cases in Egypt (2013–2050) and causes of the increase in cases.

During the period 2013–2050, population of Egypt is expected to increase to approximately 160% the 2013 population size. Applying the current age-specific incidence rates to successive populations would lead to a progressive increase in number of incident cases from 114,985 in 2013 to 331,169 in 2050, approximately 290% of 2013 incidence (Table 9 and Figure 7). This increase reflected both population growth and demographic change mainly due to ageing of population. Population growth alone would increase the number of incident cases by 55.2% in 2015. This fraction progressively decreased to become 32.8% in 2050. The fraction due to ageing gradually increased to reach 67.2% in 2050 (Figure 8).

## 4. Discussion

When the National Cancer Registry Program of Egypt was designed, there had been a number of challenges that were addressed. One of the most important challenges was design of a sample that could be representative of such a big country. Other challenges included complete capture of incident cancer cases among residents of selected governorates and best guarantee of quality of data and sustainability of the program to avoid going through survey-like data collection that need to be repeated, a methodology that proved to be inappropriate [27].

Dealing with population-based registration, the word national might be confusing. Actually, and with the exception of very few examples mainly old registries like that of Denmark and registries in small countries like Singapore, complete national coverage is not accomplished [27, 28]. The US is an example of a huge country with 18 population-based registries spread over the states without a central national registry [29]. Recently, national incidence rates of Turkey were published based on results of 8 geographically spread peripheral registries [30].

With this in mind, Egypt was geographically stratified for registration purposes into 3 regions: Lower Egypt, to the north of Cairo, and Middle, and Upper Egypt to the south. Three governorates were selected from the 3 regions representing 20.9% of the total population of Egypt. The greater Cairo, including the capital city, and Alexandria, the second capital (approximately 18 million population), were not included in the program due to difficulty in data collection and coverage of a population that daily migrates in and out of these 2 regions. Other regions that were not covered were frontier governorates; namely Matrooh, New Valley, Red Sea, and Sinai, together representing 3.3% of total population [26]. This exclusion was due to logistic reasons being mainly inhabited by nomads or internal migrants working in the tourist industry.

To ensure complete coverage of incident cancer patients in the selected governorates, data were actively collected from medical records of oncology centers within the governorates and from national referral centers like National Cancer Institute in Cairo. Registration covered health-insured patients and patients treated on government-subsidized treatment program would minimize under registration considered a point of strength of the registry. Also, regular check of death registers helped to decrease the possibility of under registration. Death certificate only cases (DCO) accounted for almost 8% of cases which could be considered an acceptable level for a newly established registry [28].

One of the elements that contributed to the National Cancer Registry Program of Egypt was the use of a unique national identification number for duplicate elimination to prevent over registration, which is a serious threat to population-based registries [7, 28]. A major concern during the development of a cancer registry especially population-based, is its sustainability. An alternative that was applied in Europe and the US was to conduct successive cancer surveys as with the US Health Interview and Examination surveys. Evaluation of these surveys was negative and this method was not recommended [28]. The main reason for lack of sustainability is unavailability of needed funding and lack of collaboration of treating physicians [28]. From the very beginning, the national cancer registry was planned to be a national program and not a project with start and end dates. During all stages of its development it was gradually incorporated within the infrastructure of the health system to gradually become part of everyday routine work [31]. Quality of data was assured through computer validation and regular manual checks on the peripheral and central levels.

We also developed a mathematical model to apply the regional age-specific incidence rates to the corresponding population structure to get national estimates of all cancers and for individual cancer sites for any specified year. Mathematical modeling is a common practice used to estimate national rates from statistics of regional registries. Recently, a model was developed for china to get national rates based upon regional registries [32]. The advantage of our Egyptian model was use of Egyptian national population-based data without importing data of adjacent countries [33].

Nonetheless, the program has its points of weakness. The data should be carefully interpreted in view of the short initial phase of registration that would be of better accuracy on successive years [28]. Furthermore, some private patients that are not covered by the Government-Subsidized Treatment Program might escape registration. This would be assumed to be a small proportion. With the rising cost of cancer treatment, most patients would seek government financial support which is a constriction right.

The results given in the current report are the first ever published incidence rates on a national and regional level for Egypt derived from a population-based cancer registry program. These incidence rates would replace the proportions that have always been derived from hospital-based results [1–5]. The results were those of Damietta (Lower Egypt), Minya (Middle Egypt), and Aswan (Upper Egypt). Damietta was selected to represent the Nile delta instead of Gharbiah registry that stopped its activities before establishment of the NCRP and needed some time to be restructured to join the program. Results of these governorates were used to compute regional rates that were used to get a national estimate based upon age-specific incidence rates and population structure.

The estimated incidence rates showed differences between the 3 regions that were mainly in the incidence of liver and bladder cancers. Among males, the proportion of incident liver cancer was highest in Lower Egypt (41.7%) and next in Middle Egypt (20.4%) and lowest in Upper Egypt (11.8%). Bladder cancer ranked next to liver cancer in Lower Egypt (8.8%). In Middle Egypt, the proportion was 14.2%, still lower than liver cancer. In Upper Egypt, liver cancer was still the most common cancer, with a small difference from bladder cancer (12.6%). These differences could be attributed to the high prevalence of hepatitis C viral infection (HCV), which is one of the highest prevalence rates worldwide [34, 35]. The distribution of liver cancer in the 3 regions followed the distribution of HCV, which is more frequent in Nile delta with decreasing prevalence going south [34]. The main risk factor for bladder cancer in Egypt was urinary Schistosomiasis which was more frequent in Upper Egypt and its prevalence decreased when going north [34]. Despite control of Schistosomiasis, its effect on bladder cancer needs time to disappear. Another difference between the 3 regions was the proportion of non-Hodgkin lymphoma that was distributed in a pattern similar to that of liver cancer [36, 37].

Among females, the pattern in Lower, Middle, and Upper Egypt was dominated by the high frequency of breast cancer (33.8%, 26.8% and 38.7% resp.) and liver cancer (16.4%, 8.9% and 5.1% resp.). This pattern of liver cancer was similar to that of males with similar relation to the prevalence of HCV. Other differences in site distribution between the 3 regions will be detailed in a separate publication [38].

The national age standardized incidence rates for all cancers in Egypt, excluding nonmelanoma skin cancer, were 175.9/100,000 for males, 157.0/100,000 for females, and 166.6/100,000 for both sexes. The age-standardized rates were intermediate between the rates of more and less developed countries [33, 39, 40]. For both sexes, the rates for all cancers excluding nonmelanoma skin cancer were 268.3/100,000 for more developed countries and 147.7/100,000 for less developed countries compared to 166.6/100,000 in Egypt. For males, the age-standardized rates were 308.7/100,000 for more developed countries and 163.0/100,000 for less developed countries compared to 175.9/100,000 in Egypt. The rates of females were 240.6/100,000 in more developed countries and 135.8/100,000 in less developed countries compared to 157.0/100,000 for Egypt.

The model used in our study revealed the seriousness of the liver cancer that ranked first among cancers in males (33.6%) and next to breast cancer. Among females the proportion of breast cancer was 32.0% followed by liver cancer (13.5%). The high prevalence of HCV especially genotype IV would explain this high incidence [34, 35]. The age-standardized rates for liver cancer for both genders were 5.4/100,000 in more developed countries, 12.0/100,000 in less developed countries compared to 43.6/100,000 in Egypt. Among males the rates were 8.6/100,000 in more developed countries, 17.8/100,000 in less developed countries compared to 61.8/100,000 in Egypt. Among females, the rates were 2.7/100,000 (more developed), 6.6/100,000 (less developed), and 24.4/100,000 (Egypt).

Incidence rate of bladder cancer that ranked next to liver cancer in males was also high. The age-standardized rates are 16.9/100,000 (more developed) and 5.3/100,000 (less developed) compared to 21.1/100,000 in Egypt. Breast cancer was the most frequent cancer among females. The age-standardized rates are 74.1/100,000 (more developed), 31.3/100,000 (less developed), and 48.8/100,000 (Egypt). Analysis of individual sites of cancers will be detailed in a separate publication [38].

Applying the model over successive years for the projected populations showed an increase in number of incident cases from approximately 115,000 patients in 2013 to more than 331,000 in 2050, almost 3-fold increase. The fraction of increase due to population growth gradually decreased over the years with a corresponding increase in the fraction due to demographic transition with ageing of the population. The cancer problem in Egypt would thus be expected to continue simply due to the inevitable ageing of the population with better standards of health care (Table 10).

Rates estimated by the model developed for the study were based on certain assumptions that should be considered in interpretation of results. These assumptions were: (a) constant age-specific rates during the study period; (b) populations of Greater Cairo and Alexandria would be considered a mosaic of the 3 registration regions and would affect the number of incident cases, and not rates; and (c) exclusion of frontier governorates (3.3% of total population of Egypt) that need special studies to get reliable cancer statistics.

## 5. Conclusion

These results are the first ever published incidence rates for Egypt on a national and regional level and clearly demonstrated the seriousness of the cancer problem of Egypt with age-adjusted incidence rates approaching those of the more developed countries. Liver cancer is a serious if not the most serious cancer problem in Egypt. Nonetheless, these rates should be carefully interpreted being based on a short initial phase of registration and a mathematical model that used regional incidence rates.

## Figures and Tables

**Table 1:** Population parameters of the 3 regions of Egypt and corresponding registries used in the current report to calculate regional and national incidence rates.

Region characteristics	Upper Egypt	Middle Egypt	Lower Egypt
Regional Registry	Aswan	Minia	Damietta
Registration period	2008	2009	2009–2011
Registry Population	1,074,131	4,426,528	3,586,056∗∗
Region population∗	4,645,449	16,161,200	30,342,291

*Based upon 2006 census.

**Person years during the registration period.

**Table 2:** Incidence rates of Cancer in Egypt (/100,000 populations) classified by region and sex for all cancer sites with and without nonmelanoma skin cancer (C44).

	Males		Females		All		Male : female ratio	
	Crude rate	ASR	Crude rate	ASR	Crude rate	ASR	Crude rate	ASR
	(95% CI)	(95% CI)	(95% CI)	(95% CI)	(95% CI)	(95% CI)		
All sites								
(i) Upper Egypt	97.1	142.8	116.9	167.1	107.0	155.0	0.8 : 1	0.9 : 1
	(89.1–105.8)	(133.1–153.2)	(108.1–126.5)	(156.5–178.4)	(101.0–113.3)	(147.7–162.6)		
(ii) Middle Egypt	109.7	170.0	95.9	132.1	102.9	151.1	1.1 : 1	1.3 : 1
	(105.4–114.1)	(164.7–175.5)	(91.1–100.2)	(127.4–137.0)	(100.0–106.0)	(147.5–154.8)		
(iii) Lower Egypt	138.5	191.8	131.7	173.3	135.2	182.6	1.1 : 1	1.1 : 1
	(133.2–144.0)	(185.6–198.2)	(126.5–137.2)	(167.3–179.6)	(131.4–139.1)	(178.2–187.1)		
(iv) Calculated rates of Egypt	117.3	178.5	111.7	159.1	114.5	169.0	1.1 : 1	1.1 : 1
	(116.0–118.6)	(176.9–180.2)	(110.4–113.0)	(157.6–160.7)	(113.6–115.5)	(167.9–170.2)		

All sites (excluding nonmelanoma skin cancer C44)								
(i) Upper Egypt	96.0	141.0	115.1	163.9	105.5	152.5	0.8 : 1	0.9 : 1
	(88.1–1104.6)	(131.4–151.4)	(106.3–124.5)	(153.4–175.1)	(99.5–111.8)	(145.5–160.1)		
(ii) Middle Egypt	108.0	167.2	94.9	130.7	101.6	149.0	1.1 : 1	1.3 : 1
	(103.8–112.3)	(162.0–172.6)	(90.9–99.1)	(126.0–135.6)	98.7–104.6)	(145.5–152.6)		
(iii) Lower Egypt	136.7	189.1	130.1	170.9	133.5	180.0	1.1 : 1	1.1 : 1
	(131.5–142.2)	(182.9–195.5)	(124.8–135.5)	(164.9–177.1)	(129.7–137.3)	(175.7–184.4)		
(iv) Calculated rates of Egypt	115.7	175.9	110.3	157.0	113.1	166.6	1 : 1	1.1 : 1
	(114.4–117.0)	(174.3–177.5)	(109.0–111.6)	(155.4–158.5)	(112.2–114.0)	(165.5–167.8)		

**Table 3:** Calculated age-specific incidence rates, crude rates, and ASR (World), males, Egypt 2008–2011.

Site	0–4	5–9	10–14	15–19	20–24	25–29	30–34	35–39	40–44	45–49	50–54	55–59	60–64	65–69	70–74	75+	Crude rate	ASR	%
Lip	—	—	—	—	—	—	0.5	—	0.3	—	0.8	1.4	0.6	1.8	8.7	1.5	0.3	0.4	0.23%
Tongue	—	—	—	—	—	—	—	—	0.3	1.4	1.3	3.1	1.9	1.8	—	2.2	0.3	0.5	0.27%
Mouth	—	—	—	0.1	—	—	0.2	0.5	0.8	1.5	2.5	4.6	0.6	2.6	1.4	—	0.5	0.6	0.41%
Salivary glands	—	—	0.1	—	0.1	—	—	0.2	—	—	0.7	1.4	2.0	2.6	4.5	5.2	0.3	0.5	0.25%
Tonsil	—	—	—	—	—	—	—	—	0.3	—	—	—	—	—	—	—	—	—	0.01%
Other oropharynx	—	—	—	—	0.1	—	—	—	—	—	0.4	0.6	—	0.8	2.8	—	0.1	0.1	0.07%
Nasopharynx	—	—	—	—	0.3	—	—	0.2	—	—	3.4	0.4	—	3.6	—	5.2	0.3	0.4	0.26%
Hypopharynx	—	—	—	0.1	—	—	—	—	0.3	—	0.9	1.4	0.6	—	1.4	1.5	0.2	0.2	0.13%
Pharynx unspec.	—	—	—	—	—	—	0.2	—	—	1.0	—	—	—	—	—	1.5	0.1	0.1	0.07%
Esophagus	—	—	—	—	0.3	0.2	0.3	—	1.4	1.8	6.1	4.1	11.2	10.8	24.4	14.7	1.4	2.3	1.20%
Stomach	—	0.1	0.1	—	0.3	0.2	0.7	0.5	2.0	2.6	5.0	6.0	11.9	6.4	21.5	14.1	1.5	2.3	1.26%
Small intestine	0.1	—	—	—	—	—	0.5	0.5	0.6	0.3	0.4	1.4	—	0.8	—	1.5	0.2	0.3	0.17%
Colon	—	—	—	0.1	0.3	0.7	2.1	2.5	1.9	6.5	14.2	11.0	19.8	18.9	31.5	32.6	3.1	4.7	2.63%
Rectum	—	—	—	0.1	0.7	1.2	0.7	1.2	2.2	2.0	3.4	4.1	3.4	2.6	8.7	1.5	1.0	1.3	0.84%
Anus	—	—	—	—	0.3	—	0.5	0.5	0.6	0.6	—	2.1	2.0	1.0	—	—	0.3	0.3	0.24%
Liver	0.3	0.4	0.4	0.2	0.3	1.3	1.4	6.1	21.2	76.3	174.2	258.7	323.1	313.8	327.1	363.5	39.5	61.8	33.63%
Gallbladder and so forth	—	—	—	—	—	0.4	—	0.5	0.6	0.6	1.0	1.0	5.9	1.0	4.7	7.4	0.5	0.7	0.40%
Pancreas	—	—	—	—	—	—	1.3	0.2	2.5	7.7	9.0	14.9	25.3	26.4	12.7	19.3	2.7	4.2	2.31%
Nose, sinuses and so forth	—	0.1	—	—	—	—	—	0.5	0.3	—	1.2	—	—	—	1.4	8.2	0.2	0.3	0.17%
Larynx	—	—	—	—	—	—	0.2	—	2.1	2.6	6.6	6.8	18.2	11.0	23.2	32.5	1.9	3.1	1.58%
Trachea, Bronchus, Lung	0.1	—	0.1	0.4	0.8	1.2	1.2	3.0	3.8	12.5	19.3	39.1	49.0	49.3	73.9	76.1	6.7	10.4	5.69%
Other Thoracic organs	0.3	—	—	0.1	0.2	0.2	—	—	0.6	1.2	2.8	3.5	3.2	7.1	9.8	8.9	0.8	1.2	0.65%
Bone	0.1	1.2	1.4	0.7	1.8	0.8	1.0	1.0	1.4	2.0	4.2	3.7	13.9	8.6	8.4	15.6	1.9	2.5	1.62%
Melanoma of skin	—	—	—	—	0.1	0.2	—	—	0.6	—	—	0.4	—	1.8	2.8	—	0.1	0.2	0.11%
Other skin	0.6	—	0.1	—	0.3	0.4	0.5	0.5	1.1	0.6	3.2	4.5	14.0	14.2	18.5	30.4	1.6	2.6	1.35%
Mesothelioma	—	—	—	—	—	—	—	—	—	0.8	0.5	1.4	—	0.8	—	—	0.1	0.2	0.10%
Kaposi sarcoma	—	—	—	—	—	—	0.2	—	—	—	1.1	—	1.3	0.8	1.4	—	0.1	0.2	0.10%
Connective, Soft tissue	0.3	0.1	0.1	0.6	1.1	2.0	1.3	1.3	3.1	3.5	3.6	8.3	4.6	6.1	18.8	2.2	1.7	2.2	1.47%
Breast	—	—	—	—	—	0.4	—	0.3	2.2	0.3	2.5	3.1	3.4	3.6	2.8	5.9	0.6	0.9	0.51%
Penis	—	—	—	—	—	—	—	—	—	—	—	—	—	—	—	—	—	—	0.00%
Prostate	—	—	—	—	—	0.2	0.3	—	0.3	1.3	4.1	11.2	24.6	47.5	90.3	216.5	5.0	9.3	4.27%
Testis	—	0.1	—	—	1.1	1.2	0.7	1.5	0.6	0.6	0.4	0.4	0.6	—	1.4	2.2	0.5	0.5	0.43%
Other male genital	—	—	—	—	—	—	—	—	—	—	0.4	—	—	0.8	1.4	—	—	0.1	0.04%
Kidney	1.2	0.1	—	—	—	0.2	0.2	0.5	0.5	3.4	6.9	9.8	7.9	16.9	14.1	22.3	1.8	2.7	1.53%
Renal pelvis	—	—	—	—	0.1	—	—	0.2	0.6	0.3	0.4	1.9	2.0	1.8	2.8	4.4	0.3	0.4	0.25%
Ureter	—	—	—	—	—	—	—	—	—	—	—	—	—	—	1.4	3.0	—	0.1	0.04%
Bladder	0.1	0.1	—	0.3	0.1	1.2	1.7	2.2	4.0	9.2	28.7	68.4	97.2	128.6	194.8	205.6	12.6	21.1	10.71%
Other urinary organs	—	—	—	—	—	—	—	—	—	—	—	—	0.6	—	—	—	—	—	0.01%
Eye	0.5	0.3	—	—	—	—	—	—	—	0.4	0.4	1.8	0.6	—	—	—	0.2	0.2	0.16%
Brain, Nervous tissue	2.9	3.0	1.8	1.7	1.5	3.3	4.2	6.8	7.3	7.3	15.5	14.5	27.2	30.4	46.7	91.4	6.4	9.0	5.48%
Thyroid	—	—	—	—	0.2	0.8	1.2	1.9	1.1	3.2	4.5	4.3	6.6	8.7	3.0	—	1.1	1.5	0.95%
Adrenal gland	0.3	0.1	—	—	—	—	—	—	—	—	—	0.6	—	0.8	1.4	—	0.1	0.1	0.08%
Other endocrine	0.5	—	0.1	—	0.3	—	—	0.5	0.3	1.0	0.4	1.3	0.6	—	2.8	—	0.3	0.4	0.25%
Hodgkin disease	0.5	1.7	1.7	1.5	1.5	1.4	—	0.2	1.7	2.5	2.0	1.4	5.3	6.1	2.8	1.5	1.5	1.7	1.29%
Non-Hodgkin lymphoma	1.1	1.2	1.5	1.0	2.2	2.5	2.1	3.6	5.8	16.6	24.4	36.0	33.8	29.5	38.1	28.8	6.4	8.8	5.48%
Immunoproliferative dis.	—	—	—	—	—	—	—	—	—	—	—	—	—	—	—	—	—	—	0.00%
Multiple myeloma	—	—	—	—	—	—	0.5	0.5	1.1	0.4	3.1	3.9	8.0	0.8	2.8	2.9	0.6	0.9	0.53%
Lymphoid leukemia	3.9	2.2	0.7	0.2	1.4	—	0.7	1.5	1.1	1.8	1.5	5.0	6.0	8.9	5.6	—	1.8	2.1	1.50%
Myeloid Leukemia	0.8	0.4	0.3	0.8	1.0	0.4	1.1	0.7	1.4	1.8	4.2	3.3	3.4	2.6	4.5	1.5	1.1	1.3	0.96%
Leukemia unspec.	1.0	1.2	1.1	0.6	2.1	2.2	1.7	0.5	2.1	4.7	1.8	6.0	8.0	3.5	7.3	20.8	2.1	2.6	1.80%
Other & unspecified	2.4	2.8	0.9	1.7	1.3	1.8	3.3	3.1	6.1	15.0	22.9	25.2	44.9	54.7	64.7	83.6	7.6	11.2	6.52%

All sites Total	17.1	15.4	10.5	10.5	20.1	23.9	30.6	42.8	83.8	195.4	389.6	582.4	793.5	840.2	1096.1	1335.6	117.3	178.5	100.00%
All sites but C44^#^	16.6	15.4	10.4	10.5	19.8	23.5	30.0	42.4	82.7	194.9	386.4	577.9	779.5	826.0	1077.5	1305.3	115.7	175.9	98.65%

^#^Incidence rates do not include nonmelanoma skin cancer.

**Table 4:** Calculated age-specific incidence rates, crude rates, and ASR (World), females, Egypt 2008–2011.

Site	0–4	5–9	10–14	15–19	20–24	25–29	30–34	35–39	40–44	45–49	50–54	55–59	60–64	65–69	70–74	75+	Crude rate	ASR	%
Lip	—	—	—	—	—	—	—	—	0.3	1.4	0.8	0.5	1.6	0.8	1.3	4.7	0.2	0.4	0.21%
Tongue	—	—	—	—	—	0.5	—	—	—	0.4	0.4	1.2	0.7	—	—	15.4	0.3	0.5	0.26%
Mouth	—	0.1	0.1	—	—	—	—	—	0.3	1.0	0.8	0.5	1.4	2.9	6.1	2.7	0.3	0.5	0.28%
Salivary glands	—	—	0.3	—	—	—	—	—	—	0.7	—	2.9	0.7	2.9	—	—	0.2	0.3	0.18%
Tonsil	—	—	—	—	—	—	—	—	—	—	—	—	—	2.2	—	1.4	—	0.1	0.04%
Other oropharynx	—	—	—	—	—	—	—	—	—	0.3	—	0.7	—	0.8	—	—	—	0.1	0.04%
Nasopharynx	0.1	—	0.1	—	—	—	—	—	—	—	—	—	1.6	—	—	—	0.1	0.1	0.06%
Hypopharynx	—	—	—	—	—	0.2	—	0.4	0.3	1.0	0.9	0.5	—	1.1	—	—	0.2	0.2	0.16%
Pharynx unspec.	—	—	—	—	—	0.2	—	—	—	—	—	—	—	—	—	—	—	—	0.01%
Esophagus	—	—	—	—	—	0.2	—	—	2.0	1.3	0.8	3.7	11.1	10.6	9.2	5.4	0.9	1.5	0.79%
Stomach	—	0.1	—	—	—	—	2.0	1.4	3.1	5.1	5.7	5.1	9.7	16.3	18.7	14.8	1.8	2.7	1.65%
Small intestine	—	—	—	—	—	—	—	0.4	—	1.9	1.7	—	1.4	5.2	4.8	—	0.4	0.5	0.32%
Colon	—	—	—	—	0.5	0.5	1.0	2.4	2.9	7.9	9.4	7.9	10.4	25.2	16.2	29.4	2.5	3.8	2.28%
Rectum	—	—	—	—	0.1	1.1	1.2	0.7	1.9	1.7	1.9	2.9	3.0	3.9	4.4	5.4	0.8	1.1	0.72%
Anus	—	—	—	—	—	0.2	—	—	0.3	—	0.5	—	1.6	—	1.3	—	0.1	0.1	0.09%
Liver	1.0	—	0.5	—	—	0.9	2.0	3.3	12.5	25.2	43.2	89.3	129.3	143.9	167.9	150.4	15.1	24.4	13.54%
Gallbladder and so forth	—	—	—	—	0.3	—	0.5	0.5	0.3	1.3	2.5	3.3	2.2	3.7	6.9	6.0	0.6	0.9	0.55%
Pancreas	—	—	—	—	—	0.4	—	0.5	0.6	3.0	3.4	8.6	9.0	18.7	24.4	19.5	1.6	2.6	1.41%
Nose, sinuses and so forth	0.1	—	—	—	—	—	—	—	0.3	0.7	1.2	1.2	5.4	—	—	—	0.3	0.4	0.23%
Larynx	—	—	—	—	—	—	—	—	0.3	1.0	2.0	—	3.0	—	3.0	2.1	0.3	0.4	0.23%
Trachea, Bronchus, Lung	0.7	—	0.1	0.1	0.5	2.0	1.0	1.5	1.7	6.9	12.3	11.7	15.1	11.3	35.9	38.0	3.0	4.5	2.70%
Other Thoracic organs	—	—	—	—	—	—	—	—	0.8	1.7	1.6	2.6	1.6	3.0	1.3	10.7	0.5	0.7	0.42%
Bone	0.7	0.1	1.1	2.0	1.4	0.7	0.7	2.2	0.9	2.3	6.5	6.7	12.0	4.5	2.6	16.9	2.0	2.5	1.80%
Melanoma of skin	—	—	—	—	—	—	—	—	0.6	—	—	—	—	2.2	4.4	—	0.1	0.2	0.10%
Other skin	0.3	—	—	0.2	—	0.4	0.5	—	2.0	2.7	1.2	8.6	10.4	14.0	14.9	11.8	1.4	2.2	1.24%
Mesothelioma	—	—	—	—	—	—	—	—	—	—	1.2	0.5	3.0	1.1	4.8	—	0.2	0.3	0.17%
Kaposi sarcoma	—	—	—	—	—	—	—	—	—	—	—	—	—	0.8	—	—	—	—	0.01%
Connective, Soft tissue	1.2	0.3	0.7	0.4	1.5	2.1	1.7	1.2	2.0	2.0	2.9	2.4	6.2	3.9	3.1	13.4	1.6	1.9	1.42%
Breast	—	—	—	—	3.1	9.5	29.2	49.0	86.0	106.2	136.0	172.9	177.4	166.3	138.7	148.6	35.8	48.8	32.04%
Vulva	0.1	—	0.1	—	—	—	—	—	—	0.3	—	1.0	—	0.8	—	1.4	0.1	0.1	0.09%
Vagina	—	—	—	—	—	—	—	—	—	—	—	1.0	3.0	3.3	—	1.4	0.2	0.3	0.14%
Cervix Uteri	—	—	—	—	—	0.2	0.2	—	0.6	3.6	6.5	9.1	8.3	10.5	1—	9.4	1.3	2.0	1.17%
Corpus Uteri	—	—	—	—	—	—	0.5	0.2	0.6	1.3	0.7	8.1	4.3	3.0	3.0	12.1	0.7	1.1	0.62%
Uterus unspec.	—	—	—	0.5	1.2	0.4	0.7	0.5	1.8	1.6	11.1	9.5	21.5	20.4	31.4	21.2	2.5	3.9	2.27%
Ovary	—	—	0.5	0.4	0.8	1.5	1.5	3.3	8.7	17.2	20.3	24.0	19.6	17.2	2—	26.3	4.6	6.3	4.12%
Other female genital	—	—	—	—	—	—	—	0.3	—	—	—	—	—	—	1.3	2.1	0.1	0.1	0.05%
Placenta	—	—	—	—	—	0.4	—	—	—	—	—	—	—	—	—	—	—	—	0.03%
Kidney	1.5	0.1	—	—	—	0.5	0.8	0.5	0.6	2.9	2.3	1.0	10.4	10.7	1.3	7.3	1.1	1.6	0.97%
Renal pelvis	—	0.3	—	—	—	0.6	—	0.2	—	0.7	0.4	0.5	1.6	0.8	—	—	0.2	0.3	0.19%
Ureter	—	—	—	—	—	—	—	—	—	—	—	—	—	—	—	—	—	—	0.00%
Bladder	0.1	—	—	0.2	—	0.2	—	0.7	0.8	5.3	8.2	18.6	20.2	37.8	43.6	53.9	3.3	5.5	2.96%
Other urinary organs	—	—	—	—	—	—	—	—	—	—	—	—	—	2.2	—	—	—	0.1	0.03%
Eye	0.1	0.3	—	—	—	—	0.5	—	—	—	—	1.2	0.7	0.8	—	1.4	0.2	0.2	0.14%
Brain, Nervous tissue	3.3	2.1	1.9	0.8	2.9	0.7	4.5	6.5	6.6	9.5	11.3	12.1	31.4	26.3	33.1	72.2	5.8	8.0	5.18%
Thyroid	—	—	—	—	2.7	2.8	10.2	7.2	6.4	9.7	10.2	11.0	9.0	2.6	10.4	14.2	3.7	4.3	3.28%
Adrenal gland	1.5	0.1	—	—	—	—	—	—	—	—	—	1.2	—	—	—	1.4	0.2	0.3	0.20%
Other endocrine	—	0.1	—	0.1	0.1	0.3	—	0.5	0.3	0.3	0.4	1.0	0.7	0.8	—	—	0.2	0.2	0.19%
Hodgkin disease	—	0.6	0.4	1.9	1.3	0.4	1.2	0.7	0.6	0.4	—	1.2	3.8	—	—	—	0.8	0.8	0.70%
Non-Hodgkin lymphoma	0.9	0.7	—	1.1	0.8	1.2	1.7	3.3	3.8	8.6	16.0	23.4	24.1	16.8	31.3	36.7	4.2	6.1	3.80%
Immunoproliferative dis.	—	—	—	—	—	—	—	—	—	—	—	—	—	—	—	—	—	—	0.00%
Multiple myeloma	—	—	—	—	—	—	—	—	0.6	1.4	0.8	1.2	2.3	4.4	6.1	2.7	0.4	0.6	0.34%
Lymphoid leukemia	2.0	0.7	0.9	0.4	0.5	—	0.7	0.7	1.4	1.0	2.7	1.7	2.8	3.9	4.4	—	1.0	1.2	0.93%
Myeloid Leukemia	0.5	0.3	0.7	0.2	1.5	1.5	1.4	1.0	0.9	0.7	4.0	3.1	6.8	7.5	—	4.9	1.3	1.6	1.14%
Leukemia unspec.	0.4	0.7	0.7	1.3	0.9	1.7	1.5	0.7	1.4	2.7	3.3	5.5	6.1	8.3	7.4	14.8	1.7	2.2	1.55%
Other & unspecified	1.4	0.9	1.9	1.7	2.4	3.9	4.6	5.5	9.6	12.5	23.8	37.3	39.9	42.1	42.6	70.5	7.7	10.8	6.93%

All sites Total	16.1	7.8	10.1	11.4	22.4	34.9	69.7	95.2	163.7	255.3	359.1	506.5	633.7	665.8	715.9	850.5	111.7	159.1	100.00%
All sites but C44^#^	15.8	7.8	10.1	11.2	22.4	34.6	69.3	95.2	161.6	252.5	357.9	497.8	623.3	651.8	701.0	838.7	110.3	157.0	98.76%

^#^Incidence rates do not include nonmelanoma skin cancer.

**Table 5:** The most frequent cancers in Egypt estimated using the results of the National Population-Based Registry Program of Egypt 2008–2011.

	Site	%	Crude rate	ASR
Males	Liver	33.63	39.5	61.8
	Bladder	10.71	12.6	21.1
	Lung^#^	5.69	6.7	10.4
	Non-Hodgkin lymphoma	5.48	6.4	8.8
	Brain^##^	5.48	6.4	8.8
	Prostate	4.27	5.0	9.3

Females	Breast	32.04	35.8	48.8
	Liver	13.54	15.1	24.4
	Brain^##^	5.18	5.8	8.0
	Ovary	4.12	4.6	6.3
	Non-Hodgkin lymphoma	3.80	4.2	6.1
	Thyroid	3.28	3.7	4.3

Both Sexes	Liver	23.81	27.5	43.6
	Breast	15.41	17.8	24.3
	Bladder	6.94	8.0	13.5
	Brain^##^	5.29	6.1	8.5
	Non-Hodgkin lymphoma	4.64	5.4	7.5
	Lung^#^	4.22	4.9	7.5

^#^Includes trachea, bronchus, and lung tumors.

^##^Includes brain and nervous system tumors.

**Table 6:** Incidence rates/100,000 population of individual cancer sites in Lower, Middle, and Upper Egypt: males.

Primary site	Lower Egypt			Middle Egypt			Upper Egypt		
	2009–2011			2009			2008		
	Crude	ASR	%	Crude	ASR	%	Crude	ASR	%
Lip	0.4	0.4	0.28%	0.3	0.5	0.24%	0.4	0.6	0.38%
Tongue	0.3	0.3	0.20%	0.2	0.4	0.20%	1.1	1.5	1.15%
Mouth	0.2	0.3	0.16%	0.8	1.1	0.73%	1.3	1.9	1.34%
Salivary glands	0.3	0.4	0.20%	0.4	0.5	0.36%	0.6	0.9	0.57%
Tonsil	0.0	0.0	0.00%	0.0	0.1	0.04%	0.0	0.0	0.00%
Other oropharynx	0.0	0.0	0.00%	0.2	0.3	0.20%	0.2	0.3	0.19%
Nasopharynx	0.5	0.5	0.35%	0.1	0.1	0.12%	0.6	1.0	0.57%
Hypopharynx	0.0	0.0	0.00%	0.3	0.5	0.28%	0.6	0.9	0.57%
Pharynx unspec.	0.1	0.1	0.04%	0.1	0.1	0.08%	0.2	0.3	0.19%
Esophagus	1.0	1.3	0.71%	1.8	3.0	1.61%	3.9	5.5	4.01%
Stomach	1.4	2.0	0.98%	1.7	2.5	1.53%	2.4	3.8	2.48%
Small intestine	0.1	0.1	0.04%	0.5	0.6	0.44%	0.2	0.2	0.19%
Colon	4.0	5.4	2.91%	2.3	3.7	2.10%	2.4	3.7	2.48%
Rectum	0.9	1.1	0.67%	1.2	1.6	1.13%	0.7	1.1	0.76%
Anus	0.4	0.4	0.28%	0.2	0.2	0.16%	0.4	0.5	0.38%
Liver	57.8	81	41.71%	22.4	37.6	20.42%	11.5	17.5	11.83%
Gallbladder and so forth	0.5	0.5	0.39%	0.4	0.7	0.32%	1.3	2.2	1.34%
Pancreas	3.2	4.4	2.28%	2.1	3.5	1.94%	3.5	5.4	3.63%
Nose, sinuses and so forth	0.1	0.1	0.04%	0.4	0.6	0.32%	0.6	0.9	0.57%
Larynx	0.8	1.3	0.59%	3.3	5.7	3.03%	3.9	6.0	4.01%
Trachea, Bronchus, Lung	7.6	10.1	5.47%	6.3	10.8	5.77%	7.4	11.5	7.63%
Other Thoracic organs	1.0	1.3	0.71%	0.8	1.2	0.69%	0.6	0.8	0.57%
Bone	1.9	2.4	1.34%	2.3	3.4	2.10%	1.5	1.9	1.53%
Melanoma of skin	0.2	0.2	0.12%	0.1	0.1	0.12%	0.0	0.0	0.00%
Other skin	1.8	2.7	1.30%	1.7	2.8	1.53%	1.1	1.8	1.15%
Mesothelioma	0.0	0.0	0.00%	0.3	0.4	0.24%	0.4	0.6	0.38%
Kaposi sarcoma	0.1	0.1	0.08%	0.2	0.3	0.16%	0.0	0.0	0.00%
Connective, Soft tissue	2.5	2.6	1.77%	0.5	0.8	0.48%	3.0	3.8	3.05%
Breast	0.7	0.8	0.47%	0.4	0.6	0.32%	1.1	1.8	1.15%
Penis	0.0	0.0	0.00%	0.0	0.0	0.00%	0.0	0.0	0.00%
Prostate	6.7	11.7	4.84%	2.9	5.2	2.66%	5.7	9.2	5.92%
Testis	0.5	0.4	0.35%	0.5	0.7	0.48%	0.4	0.5	0.38%
Other male genital	0.0	0.0	0.00%	0.1	0.2	0.12%	0.0	0.0	0.00%
Kidney	2.2	3.2	1.61%	1.7	2.5	1.53%	0.9	1.3	0.95%
Renal pelvis	0.2	0.3	0.12%	0.5	0.8	0.48%	0.4	0.5	0.38%
Ureter	0.1	0.1	0.04%	0.0	0.1	0.04%	0.0	0.0	0.00%
Bladder	12.2	19	8.82%	15.6	26.4	14.25%	12.2	19.3	12.60%
Other urinary organs	0.0	0.0	0.00%	0.0	0.0	0.00%	0.2	0.3	0.19%
Eye	0.1	0.2	0.08%	0.3	0.4	0.24%	0.7	0.6	0.76%
Brain, Nervous tissue	6.2	8.1	4.49%	8.0	12.5	7.26%	5.2	6.7	5.34%
Thyroid	1.3	1.5	0.91%	1.2	1.7	1.05%	0.7	1.1	0.76%
Adrenal gland	0.0	0.0	0.00%	0.1	0.2	0.12%	0.6	0.8	0.57%
Other endocrine	0.1	0.1	0.08%	0.6	0.8	0.56%	0.4	0.4	0.38%
Hodgkin disease	1.6	1.8	1.18%	1.7	2.0	1.53%	1.5	1.5	1.53%
Non-Hodgkin lymphoma	8.3	10.3	6.03%	5.2	7.6	4.76%	2.8	4.2	2.86%
Immunoproliferative dis.	0.0	0.0	0.00%	0.0	0.0	0.00%	0.0	0.0	0.00%
Multiple myeloma	0.7	0.9	0.51%	0.7	1.1	0.61%	0.2	0.3	0.19%
Lymphoid leukemia	1.7	2.2	1.22%	1.8	2.3	1.61%	3.0	3.1	3.05%
Myeloid Leukemia	0.9	1.0	0.63%	1.6	1.8	1.45%	1.9	2.5	1.91%
Leukemia unspec.	2.4	3.1	1.73%	2.0	2.6	1.86%	1.1	1.1	1.15%
Other & unspecified	5.9	8.3	4.25%	13.9	17.6	12.67%	8.7	13	8.97%

All sites Total	138.5	191.8	100.00%	109.7	170	100.00%	97.1	142.8	100.00%
All sites but C44^#^	136.7	189.1	98.70%	108	167.2	98.47%	96	141	98.85%

^#^Incidence rates do not include nonmelanoma skin cancer.

**Table 7:** Incidence rates/100,000 population of individual cancer sites in Lower, Middle, and Upper Egypt: females.

Primary site	Lower Egypt			Middle Egypt			Upper Egypt		
	2009–2011			2009			2008		
	Crude	ASR	%	Crude	ASR	%	Crude	ASR	%
Lip	0.3	0.4	0.26%	0.2	0.4	0.24%	0.0	0.0	0.00%
Tongue	0.3	0.5	0.26%	0.3	0.5	0.34%	0.2	0.2	0.16%
Mouth	0.1	0.2	0.09%	0.5	0.8	0.53%	0.9	1.5	0.80%
Salivary glands	0.2	0.2	0.13%	0.3	0.4	0.29%	0.2	0.4	0.16%
Tonsil	0.1	0.1	0.04%	0.0	0.1	0.05%	0.0	0.0	0.00%
Other oropharynx	0.0	0.0	0.00%	0.1	0.1	0.10%	0.2	0.3	0.16%
Nasopharynx	0.1	0.1	0.04%	0.0	0	0.05%	0.2	0.2	0.16%
Hypopharynx	0.0	0.0	0.00%	0.4	0.4	0.38%	0.6	0.9	0.48%
Pharynx unspec.	0.0	0.0	0.00%	0.0	0.0	0.05%	0.0	0.0	0.00%
Esophagus	0.9	1.2	0.65%	1.2	1.9	1.25%	1.1	1.6	0.96%
Stomach	2.3	3.2	1.73%	1.2	1.8	1.25%	1.9	3.1	1.60%
Small intestine	0.3	0.5	0.22%	0.4	0.6	0.43%	0.6	0.9	0.48%
Colon	3.0	4.2	2.30%	2.2	3.2	2.31%	2.4	3.5	2.08%
Rectum	0.9	1.0	0.65%	1.0	1.2	1.01%	0.7	1.3	0.64%
Anus	0.1	0.1	0.04%	0.1	0.2	0.14%	0.2	0.3	0.16%
Liver	21.6	32.6	16.37%	8.6	13.7	8.95%	6.0	8.7	5.12%
Gallbladder and so forth	0.5	0.5	0.35%	0.6	0.9	0.58%	1.9	3.1	1.60%
Pancreas	2.1	3.2	1.60%	0.9	1.4	0.91%	1.7	2.3	1.44%
Nose, sinuses and so forth	0.3	0.5	0.26%	0.1	0.2	0.14%	0.2	0.2	0.16%
Larynx	0.2	0.3	0.17%	0.3	0.4	0.29%	0.4	0.7	0.32%
Trachea, Bronchus, Lung	3.7	5.3	2.82%	2.2	3.1	2.26%	2.4	3.8	2.08%
Other Thoracic organs	0.6	0.8	0.43%	0.5	0.7	0.48%	0.0	0.0	0.00%
Bone	2.0	2.3	1.52%	1.8	2.4	1.92%	3.4	4.4	2.88%
Melanoma of skin	0.2	0.3	0.17%	0.0	0.1	0.05%	0.0	0.0	0.00%
Other skin	1.7	2.4	1.26%	1.0	1.5	1.06%	1.9	3.1	1.60%
Mesothelioma	0.3	0.3	0.22%	0.2	0.3	0.24%	0.4	0.7	0.32%
Kaposi sarcoma	0.0	0.0	0.00%	0.0	0.1	0.05%	0.0	0.0	0.00%
Connective, Soft tissue	2.3	2.6	1.78%	0.4	0.6	0.38%	1.9	2.2	1.60%
Breast	43.8	53	33.22%	25.8	35.6	26.84%	45.3	64.5	38.72%
Vulva	0.0	0.0	0.00%	0.3	0.4	0.34%	0.0	0.0	0.00%
Vagina	0.1	0.2	0.09%	0.1	0.2	0.14%	0.6	1.0	0.48%
Cervix Uteri	1.7	2.4	1.26%	1.0	1.5	1.06%	0.6	0.9	0.48%
Corpus Uteri	0.6	0.9	0.43%	0.6	0.9	0.67%	1.7	2.9	1.44%
Uterus unspec.	3.7	5.3	2.77%	1.0	1.3	1.06%	2.4	3.8	2.08%
Ovary	5.1	6.4	3.90%	3.6	5.0	3.75%	7.1	10.2	6.08%
Other female genital	0.0	0.0	0.00%	0.0	0.1	0.05%	0.4	0.6	0.32%
Placenta	0.0	0.0	0.00%	0.0	0.0	0.05%	0.2	0.2	0.16%
Kidney	1.1	1.6	0.87%	1.2	1.8	1.25%	0.7	1.1	0.64%
Renal pelvis	0.2	0.3	0.17%	0.2	0.3	0.19%	0.2	0.2	0.16%
Ureter	0.0	0.0	0.00%	0.0	0.0	0.00%	0.0	0.0	0.00%
Bladder	3.7	5.9	2.77%	3.1	4.9	3.27%	3.6	5.7	3.04%
Other urinary organs	0.1	0.1	0.04%	0.0	0.0	0.00%	0.0	0.0	0.00%
Eye	0.1	0.1	0.04%	0.3	0.5	0.34%	0.2	0.2	0.16%
Brain, Nervous tissue	5.8	7.4	4.42%	7.4	11.1	7.70%	2.4	2.9	2.08%
Thyroid	5.1	5.4	3.90%	1.6	2.1	1.64%	3.6	4.2	3.04%
Adrenal gland	0.2	0.3	0.17%	0.2	0.3	0.24%	0.2	0.2	0.16%
Other endocrine	0.1	0.0	0.04%	0.5	0.7	0.53%	0.2	0.2	0.16%
Hodgkin disease	1.0	0.7	0.74%	1.0	1.0	1.01%	0.9	0.9	0.80%
Non-Hodgkin lymphoma	5.4	6.7	4.11%	4.2	5.8	4.43%	2.6	3.8	2.24%
Immunoproliferative dis.	0.0	0.0	0.00%	0.0	0.0	0.00%	0.0	0.0	0.00%
Multiple myeloma	0.7	0.9	0.52%	0.1	0.3	0.14%	0.2	0.0	0.16%
Lymphoid leukemia	1.0	1.1	0.74%	1.2	1.6	1.25%	1.5	1.6	1.28%
Myeloid Leukemia	0.9	1.1	0.65%	1.7	2.0	1.78%	2.2	3.3	1.92%
Leukemia unspec.	1.8	2.4	1.34%	1.6	2.0	1.68%	1.9	2.3	1.60%
Other & unspecified	5.9	8.2	4.46%	14.3	15.5	14.86%	9.2	12.9	7.84%

All sites Total	131.7	173.3	100.00%	95.9	132.1	100.00%	116.9	167.1	100.00%
All sites but C44^#^	130.1	170.9	98.74%	94.9	130.7	98.94%	115.1	163.9	98.40%

^#^Incidence rates do not include nonmelanoma skin cancer.

**Table 8:** Proportions and incidence rates of the most frequently observed cancers in the 3 regions of Egypt.

	Lower Egypt				Middle Egypt				Upper Egypt			
	2009–2011				2009				2008			
	Site	%	Crude rate	ASR	Site	%	Crude rate	ASR	Site	%	Crude rate	ASR
Males	Liver	41.7	57.8	81.0	Liver	20.4	22.4	37.6	Bladder	12.6	12.2	19.3
	Bladder	8.8	12.2	19.0	Bladder	14.2	15.6	26.4	Liver	11.8	11.5	17.5
	NHL	6.0	8.3	10.3	Brain^#^	7.3	8.0	12.5	Lung^##^	7.6	7.4	11.5
	Lung^##^	5.5	7.6	10.1	Lung^##^	5.8	6.3	10.8	Leukemia	6.1	6.0	6.7
	Prostate	4.8	6.7	11.7	Leukemia	4.9	5.4	6.7	Prostate	5.9	5.7	9.2

Females	Breast	33.2	43.8	53.0	Breast	26.8	25.8	35.6	Breast	38.7	45.3	64.5
	Liver	16.4	21.6	32.6	Liver	8.9	8.6	13.7	Ovary	6.1	7.1	10.2
	Brain^#^	4.4	5.8	7.4	Brain^#^	7.7	7.4	11.1	Liver	5.1	6.0	8.7
	NHL	4.1	5.4	6.7	Leukemia	4.7	4.5	5.6	Leukemia	4.8	5.6	7.2
	Thyroid	3.9	5.1	5.4	NHL	4.4	4.2	5.8	Uterus	3.5	4.1	6.7

Both Sexes	Liver	29.6	40.1	56.8	Liver	15.2	15.6	25.7	Breast	21.6	23.1	33.2
	Breast	16.1	21.7	26.9	Breast	12.4	12.8	18.1	Liver	8.2	8.8	13.1
	Bladder	5.9	8.0	12.5	Bladder	9.2	9.5	15.7	Bladder	7.4	7.9	12.5
	NHL	5.1	6.9	8.5	Brain^#^	7.5	7.7	11.8	Leukemia	5.4	5.7	7.0
	Brain^#^	4.5	6.0	7.8	Leukemia	4.8	4.9	6.2	Lung^##^	4.6	4.9	7.7

^#^Includes brain and nervous system tumors.

^##^Includes trachea, bronchus and lung tumors.

**Table 9:** Estimated number of cancer cases, Egypt 2013–2050.

	2013			2015			2020			2025			2050		
	Males	Females	Total	Males	Females	Total	Males	Females	Total	Males	Females	Total	Males	Females	Total
Lip	135	126	262	146	135	281	178	164	342	206	202	408	438	427	866
Tongue	155	175	330	164	185	349	186	231	417	219	296	515	417	763	1180
Mouth	228	163	391	243	178	420	275	216	491	314	261	575	544	528	1071
Salivary glands	147	108	255	158	117	275	189	130	319	222	153	375	495	265	760
Tonsil	6	31	37	6	33	40	9	41	50	11	54	65	13	129	142
Other oropharynx	42	25	67	45	28	73	54	30	85	63	37	100	132	65	198
Nasopharynx	145	32	178	154	34	188	171	40	211	207	42	249	428	68	496
Hypopharynx	72	80	152	76	85	161	88	96	184	102	111	213	200	173	372
Pharynx unspec.	35	7	42	36	7	43	42	7	48	55	7	62	94	8	102
Oesophagus	699	485	1184	746	525	1271	897	644	1542	1065	762	1827	2249	1504	3752
Stomach	726	969	1695	772	1045	1816	922	1249	2171	1080	1484	2565	2185	2877	5062
Small intestine	98	179	277	106	194	300	120	229	349	134	274	408	223	507	730
Colon	1522	1339	2862	1618	1437	3055	1893	1715	3608	2225	2063	4287	4465	4120	8585
Rectum	464	406	871	490	432	922	568	502	1070	645	584	1230	1097	1052	2149
Anus	133	50	183	142	53	195	162	65	227	178	71	249	291	127	418
Liver	19646	8345	27991	20932	9043	29975	24420	10900	35320	28580	12933	41513	59047	26425	85471
Gallbladder and so forth	235	324	559	248	350	598	297	413	710	348	488	835	715	967	1682
Pancreas	1350	876	2226	1440	957	2397	1676	1160	2836	1961	1405	3366	3912	2971	6883
Nose, sinuses and so forth	98	136	234	104	144	247	124	170	294	154	186	340	340	322	661
Larynx	933	134	1067	993	142	1136	1194	173	1367	1428	201	1629	3094	395	3489
Trachea, Bronchus, Lung	3304	1586	4890	3530	1703	5233	4168	2031	6198	4889	2404	7293	10176	4895	15071
Other Thoracic organs	368	260	628	393	277	670	464	335	798	551	415	966	1149	897	2046
Bone	889	957	1846	935	1011	1946	1068	1174	2242	1216	1330	2546	2212	2401	4613
Melanoma of skin	60	59	119	66	66	132	82	84	166	95	105	200	181	207	388
Other skin	797	749	1547	853	813	1666	1018	973	1990	1205	1160	2365	2641	2306	4947
Mesothelioma	55	106	161	58	116	174	65	143	207	76	161	238	135	321	456
Kaposi sarcoma	60	8	68	63	9	72	72	10	82	81	13	95	159	28	187
Connective, Soft tissue	801	751	1552	846	787	1633	981	901	1882	1113	1026	2139	1924	1796	3720
Breast	287	17905	18192	306	19105	19411	362	22320	22682	422	25793	26215	807	45243	46050
Vulva	0	52	52	0	56	56	0	63	63	0	76	76	0	146	146
Vagina	0	95	95	0	103	103	0	126	126	0	147	147	0	305	305
Cervix Uteri	0	701	701	0	752	752	0	882	882	0	1039	1039	0	2039	2039
Corpus Uteri	0	392	392	0	426	426	0	502	502	0	600	600	0	1256	1256
Uterus unspec.	0	1353	1353	0	1456	1456	0	1758	1758	0	2055	2055	0	4143	4143
Ovary	0	2288	2288	0	2434	2434	0	2830	2830	0	3311	3311	0	5957	5957
Other female genital	0	30	30	0	34	34	0	44	44	0	55	55	0	134	134
Placenta	0	14	14	0	14	14	0	14	14	0	15	15	0	18	18
Penis	0	0	0	0	0	0	0	0	0	0	0	0	0	0	0
Prostate	2544	0	2544	2747	0	2747	3398	0	3398	4295	0	4295	10785	0	10785
Testis	229	0	229	240	0	240	266	0	266	291	0	291	425	0	425
Other male genital	21	0	21	22	0	22	28	0	28	33	0	33	73	0	73
Kidney	877	561	1438	934	594	1528	1080	693	1774	1274	801	2076	2628	1487	4115
Renal pelvis	141	99	240	152	103	255	183	115	297	214	126	341	446	196	642
Ureter	22	0	22	24	0	24	31	0	31	41	0	41	111	0	111
Bladder	6362	1872	8234	6852	2038	8891	8228	2481	10709	9746	3016	12762	21783	6554	28337
Other urinary organs	8	21	29	8	23	31	10	27	37	11	35	46	22	72	94
Eye	89	82	171	92	87	180	98	96	194	104	108	212	160	195	355
Brain, Nervous tissue	3072	2933	6004	3256	3133	6389	3779	3721	7500	4390	4351	8740	8439	8450	16888
Thyroid	547	1759	2306	582	1867	2448	661	2106	2767	748	2363	3111	1298	3814	5113
Adrenal gland	43	105	149	46	109	155	52	113	166	57	121	178	102	180	282
Other endocrine	129	95	224	136	100	236	156	114	269	174	125	300	273	188	462
Hodgkin disease	647	321	968	673	336	1008	751	371	1123	837	397	1234	1245	527	1772
Non-Hodgkin lymphoma	3053	2188	5241	3223	2345	5568	3689	2755	6444	4240	3202	7442	7749	6135	13884
Immunoproliferative dis.	0	0	0	0	0	0	0	0	0	0	0	0	0	0	0
Multiple myeloma	316	200	516	334	218	552	386	264	650	432	321	753	819	644	1463
Lymphoid leukaemia	801	471	1272	841	493	1335	926	551	1477	1000	603	1604	1535	896	2431
Myeloid Leukaemia	511	614	1125	534	642	1176	595	727	1323	666	810	1476	1054	1358	2412
Leukemia unspec.	965	842	1807	1005	891	1896	1131	1027	2157	1295	1188	2483	2226	2169	4395
Other & unspecified	3692	3965	7657	3921	4232	8153	4573	4971	9543	5358	5793	11151	10582	11026	21607

All sites Total	57558	57426	114985	61288	61495	122783	71759	72496	144255	84045	84679	168723	171494	159675	331169
All sites but C44^#^	56761	56677	113438	60435	60683	121117	70742	71523	142265	82840	83519	166358	168854	157369	326223

^#^Numbers do not include nonmelanoma skin cancer.

**Table 10:** Estimated cancer incidence in the period 2013–2050 and causes of increase.

	2013	2015	2020	2025	2050
Estimated population	85294388 (100%)	88487396 (103.7%)	96260017 (112.9%)	103742157 (121.6%)	137872522 (161.6%)
Number of cases^#1^	114985	122783 (106.8%)	144255 (125.5%)	168723 (146.7%)	331169 (288.0%)
Increased cases from 2013^#2^		7798 (6.8%)	29270 (25.5%)	53738 (46.7%)	216184 (188.0%)
Increased cases due to population growth^#3^		4303	14783	24869	70880
Increased cases due to population structure change^#4^		3494	14487	28869	145304
% Increase due to population growth^#5^		55.20%	50.50%	46.28%	32.79%

N.B.

^#1^Number of expected cases depending on 2013 rates of incidence.

^#2^Number of increased cases from 2013 number of cases.

^#3^Number of increased cases (from 2013) that is attributed to increase in population number (population growth).

^#4^Number of increased cases (from 2013) that is attributed to change in population structure (aging of population) and not to population growth.

^#5^Percent of increased number of cases (from 2013) that can be attributed to population growth only (not due to change in population structure).

## Appendix

### Statistical Formulas Used in the Analysis

Step 1 (calculation of regional and national incident cases for census year 2006/sex). (1) Number of incident cancer cases *C* in age group *x*/region *i* where *r* is incidence rate and *w* is population size:

(A.1) $$\begin{array}{c} C_{xi}=r_{xig}*w_{xig}. \end{array}$$

(2) Total incident cases in region *i*: *C*_*i*_ = ∑*C*_*xi*_. (3) Total incident cases in the 3 regions *G*: *C*_*G*_ = ∑*C*_*i*_ where *I* = 1–3. (4) Incident cases in total Egypt *M*:

(A.2) $$\begin{array}{c} C_{M}=\sum C_{G}*\left(W_{M}/W_{G}\right)\;\text{where}\;\;W\;\;\text{is}\;\;2006\;\;\text{population}. \end{array}$$

(5) Calculation of national age specific incidence rates *r*_*xM*_ = ∑*C*_*xi*_/*W*_*xi*_ for the 3 regions.

Step 2 (calculation of regional and national incident cases for a given year/sex). The same procedure was followed applying *r*_*xM*_ calculated above to the population of the year under study.

Step 3 (future projections). For estimation of incident cancer in a specific year till 2050, the national age specific incidence rates by sex were applied to the corresponding population structure [41]. This estimated number would be due to population growth and demographic change with aging of the population. The fraction due to population growth could be calculated using corresponding population sizes. The remainder of increase in number would be due mainly to demographic change.

## List of Abbreviations

ASIR: Age specific incidence rate
ASR: Age standardized rate
DCO: Death certificate only
HCV: Hepatitis C virus
IARC: International Agency for Research on Cancer
ICD-10: International Statistical Classification of Diseases and related health problems, 10th revision
NCRP: National Cancer Registry Program of Egypt
NHL: Non-Hodgkin Lymphoma
SEER: Surveillance, Epidemiology, and End Results Program.
